# A retrospective comparison of the biceps femoris long head muscle structure in athletes with and without hamstring strain injury history

**DOI:** 10.1371/journal.pone.0298146

**Published:** 2024-02-26

**Authors:** Gokhan Yagiz, Meiky Fredianto, Maria Ulfa, Indira Ariani, Avika Dwi Agustin, Nami Shida, E. Whitney G. Moore, Hans-Peter Kubis

**Affiliations:** 1 Department of Kinesiology, College of Health and Human Performance, East Carolina University, Greenville, NC, United States of America; 2 Faculty of Health Sciences, Department of Physiotherapy and Rehabilitation, Amasya University, Amasya, Republic of Türkiye; 3 Faculty of Medicine and Health Sciences, Orthopaedics and Traumatology Division, Surgery Department, School of Medicine, Universitas Muhammadiyah Yogyakarta, Yogyakarta, Indonesia; 4 Department of Orthopaedic Surgery, Siloam Hospitals Yogyakarta, Yogyakarta, Indonesia; 5 Faculty of Medicine and Health Sciences, School of Medicine, Universitas Muhammadiyah Yogyakarta, Yogyakarta, Indonesia; 6 Master of Hospital Administration, Postgraduate Program, Universitas Muhammadiyah Yogyakarta, Yogyakarta, Indonesia; 7 Department of Radiology, Siloam Hospitals Yogyakarta, Yogyakarta, Indonesia; 8 Universitas Muhammadiyah Yogyakarta, Yogyakarta, Indonesia; 9 Faculty of Health Sciences, Department of Physical Therapy, Tokyo Metropolitan University, Tokyo, Japan; 10 Institute for Applied Human Physiology, School of Human and Behavioural Sciences, Bangor University, Bangor, Wales, United Kingdom; Erzurum Technical University: Erzurum Teknik Universitesi, TURKEY

## Abstract

**Introduction:**

Hamstring strain injuries (HSI) and re-injuries are endemic in high-speed running sports. The biceps femoris long head (BFlh) is the most frequently injured muscle among the hamstrings. Structural parameters of the hamstring muscle are stated to be susceptible to strain injuries at this location. This retrospective study targeted comparing the BFlh’s structural parameters between previously injured and uninjured athletes.

**Methods:**

Nineteen male athletes with previous BFlh strain injury history and nineteen athletes without former lower extremity injury history were included in this study. Fascicle length, mid-muscle belly and distal musculotendinous (MTJ) passive stiffnesses of the biceps femoris long head (BFlh) were examined via b-mode panoramic ultrasound scanning and ultrasound-based shear-wave elastography. Parameter comparisons of both legs within and between athletes with and without injury history were performed.

**Results:**

Comparison of the BFlh fascicle length between the injured leg of the injured group and the legs of the controls revealed a trend to shorter fascicle lengths in the injured leg (*p* = 0.067, *d* = -0.62). However, the mid-muscle belly passive stiffness of the BFlh was significantly higher in the injured legs (*p* = 0.009, *d* = 0.7) compared with the controls. Additionally, the distal MTJ stiffness was much higher in the previously injured legs compared with controls (*p* < 0.001, *d* = 1.6).

**Conclusions:**

Outcomes support the importance of BFlh properties related to stiffness, and fascicle length for injury susceptibility in athletes. Future prospective studies should determine whether the higher stiffness in the injured athletes is a cause or consequence of the HSI. Physical therapy and rehabilitation programmes after HSI should focus on BFlh muscle properties i.e., elasticity and fascicle length for reducing re-injury and increasing sports performance.

## 1. Introduction

Hamstring strain injuries (HSI) are common in sports with requirements for high-speed running. A further problem with HSI is the high and severe reoccurrence of HSI [1], which could indicate difficulty in rehabilitating the initial HSI effectively. HSI incidence has increased compared with earlier epidemiologic data in various sports [1]. For example, 24% of all injuries have been recorded as HSI in soccer nowadays [2]. Hence, scientists have focused on identifying risk factors and developing an optimal prevention strategy for HSI over the last two decades.

The late swing phase of running is often described as the most vulnerable time for HSI [3–5]. During the late swing phase of running, the hamstrings produce eccentric contraction to control the antagonists and decelerate the tibia [6]. At this moment, the biceps femoris long head (BFlh) undergoes the greatest elongation among the hamstrings by reaching 110% of its length [7]. Due to this elongation, HSI generally occur when the muscle fascicles can not resist the increased tensile force [3]. In particular, HSI occur frequently in the BFlh among the hamstrings [8], with BFlh distal musculotendinous junction (MTJ) injuries being more common [9], recurrent, severe and require longer rehabilitation than other HSI [9, 10].

Besides various risk factors [11], research has identified increased passive hamstring muscle stiffness [12] and shorter BFlh fascicle lengths [13] as structural risk factors for HSI [11]. However, it was suggested that the use of trigonometric equation [14] or manual linear extrapolation [15, 16] techniques overestimate the BFlh fascicle length in comparison to the extended field of view (EFOV)/panoramic ultrasound scanning technique [17]. The former techniques were used in previous retrospective studies comparing BFlh fascicle lengths between athletes with and without injury history [18–22]. Specifically, trigonometric equations techniques (+0.5 to +1.9 cm), and manual linear extrapolation technique (+0.33 cm) overestimate BFlh fascicle lengths [17]. Additionally, a further study [12] found passive muscle stiffness is a risk factor for HSI. However, an unspecific technique (free oscillation) was used that measures the passive stiffness of all muscle and tendon units of all knee flexors. Thus, this technique does not provide specific information for the BFlh, i.e., not providing specific measurements of stiffness of the MTJ and other locations of the BFlh. The recent technological advancements allow scientists to explore the passive stiffness of a specific part of individual muscles via ultrasound-based shear-wave elastography [23]. In this way, specific information on BFlh muscle stiffness in athletes who returned to play from HSI can provide new insights into the hamstring injury rehabilitation process.

To the authors’ knowledge, no retrospective study compared the BFlh fascicle length via the EFOV technique or passive stiffness of muscle belly and distal MTJ of the BFlh in athletes with and without HSI history altogether. Exploring these structural parameters of the BFlh can bring new insights into the rehabilitation and prevention strategies for HSI.

Therefore, this study aimed to compare the BFlh fascicle length via the EFOV technique, passive stiffness of the mid-belly and MTJ of the BFlh via ultrasound-based shear wave elastography between previously injured legs and uninjured contralateral legs of athletes with HSI history, and both legs of athletes without HSI history. The hypotheses of this study were considered as follows: 1) previously injured legs will have shorter fascicles in the BFlh compared to contralateral uninjured legs and both legs of the athletes without HSI due to possible decreased exertion at the beginning of rehabilitation [22], reduced involvement in sports [24], and/or neural inhibition after the HSI [24–29]; 2) previously injured legs will have higher stiffness in the BFlh after the injury compared with contralateral uninjured legs and both legs of the athletes without HSI due to possible contractures or scars in the muscle [24, 26, 30–35].

## 2. Methodology

### 2.1. Research design

This study was designed as a retrospective comparative case-control study. The injured legs of the athletes with HSI history constituted the injured group (n = 19). The uninjured legs of the athletes with HSI formed the internal control group (n = 19). Both legs of the athletes without HSI and lower extremity injuries participating in the same sports were used as the external control group (n = 38). Ethical approval was obtained from the Health Research Ethics Committee of Universitas ‘Aisyiyah Yogyakarta (Approval No. 2843/KEP-UNISA/V/2023). Before the study, participants read and signed a written informed consent according to the Declaration of Helsinki [36]. Indonesian authors (second, third, fourth and fifth authors) of this study communicated with the participants in Indonesian languages. The recruitment and data collection of the study started on the 10^th^ of May 2023 and finished on the 30^th^ of June 2023.

### 2.2. Participants

Athletes who returned to sports after an HSI were included in the injured group. In this study, athletes were defined as people partaking professionally and semi-professionally in sports. The inclusion criteria for the injured group were: a) male athlete between 18–45 years old; b) returning to pre-level sports competition after HSI in the BFlh. Criteria for the control group were: a) male athlete between 18 and 45 years old, b) being without a known lower extremity injury history, c) performing the same sports as the injured group (e.g., if one professional football player is included in the injured group, one professional football player from the same competition level participated to the study in the control group.)

### 2.3. Sample size

A priori sample size calculation was made using the F test, one-way ANOVA option in the G*Power (Version 3.1) [37] for two groups, 0.74 effect size [22], 0.05 alpha level, 0.95 statistical power. In total, a sample size of 26 was required for the study (13 previously injured players, 13 controls). However, this study compared 19 athletes for the injured group and 19 athletes for the uninjured group. Therefore, 19 injured legs were compared to 19 contralateral uninjured legs and 38 legs of the control group to reach a high statistical power. The achieved power of the study was determined as 0.96 for the fascicle length (effect size: 0.62 (injured leg vs. uninjured legs of the control group), two groups, 0.05 alpha level and 38 sample size (total legs)); the power of 0.97 for mid-muscle belly stiffness (effect size: 0.70 (injured leg vs uninjured legs of the control group), two groups, 0.05 alpha level and 38 sample size (total legs)); and power of 1.00 for the distal MTJ stiffness measurements of the BFlh (effect size: 1.6 (injured leg vs uninjured legs of the control group), two groups, 0.05 alpha level and 38 sample size (total legs)) via G*Power (Version 3.1) [37].

### 2.4. Measurement procedures

Before starting the measurements, a medical examination was performed by a medical team consisting of an orthopaedic surgeon, a medical doctor, and a physiotherapist. Physical characteristics (height (cm), body mass (kg), body mass index (BMI)), injury history, and playing history (years) of the participants were recorded. The preferred kicking leg of a ball was accepted as the dominant leg.

The BFlh fascicle length was measured via the EFOV technique via b-mode ultrasound (LOGIQ P8, General Electric Healthcare, Wauwatosa, WI). The mid-belly and distal MTJ passive muscle stiffness measurements of the BFlh were completed via ultrasound-based shear wave elastography (SWE) (LOGIQ P8, General Electric Healthcare, Wauwatosa, WI). The BFlh fascicle length and passive stiffness measurements were performed twice for each dominant and non-dominant leg by totally removing the ultrasound probe (L3-12-RS: Wideband Linear Array Probe, 2 to 11 MHz, width: 51.2 mm, depth: 40 to 80 mm) from the skin between the measurements on the same measurement session. The average results of the two measurements were accepted as the outcomes. Reliability analyses were performed for the outcomes of BFlh fascicle length, mid-belly and distal MTJ passive stiffnesses of the BFlh between these two measurements for all the legs of all the athletes. The measurements were performed by the first author, who is highly experienced with the BFlh fascicle length, passive muscle stiffness and morphology measurements of the BFlh [16, 38, 39], alongside at least one of the medical team. The fascicle length digitisations were directly performed using the ultrasound machine’s measure function, and the results were recorded during the measurement sessions. Likewise, the ultrasound machine directly provided passive muscle stiffness results, which were recorded during the measurement sessions.

#### 2.4.1. The BFlh fascicle length measurement via the EFOV technique

**Fig 1:**
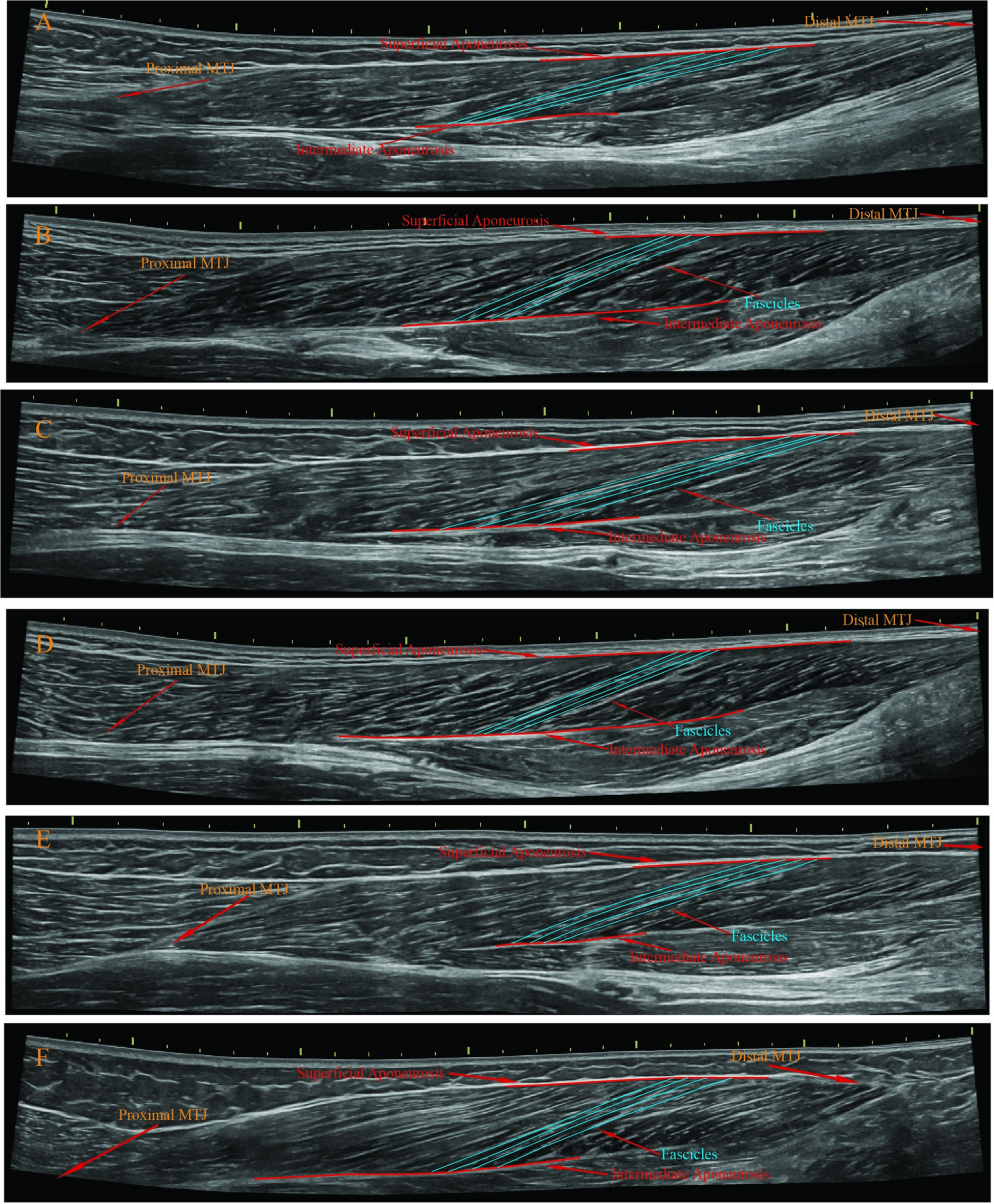
A-F: Random examples of the biceps femoris long head fascicle length measurements via panoramic ultrasound scanning in various participants.

The participants laid down on a standard medical bed in a prone position when the hamstring was at the passive resting position. Firstly, the proximal and distal MTJ were determined by monitoring via the ultrasound probe [17]. Then, the path of the BFlh muscle belly was monitored [17]. Afterwards, the ultrasound probe was continuously moved parallel to the BFlh muscle orientation, starting from the distal MTJ towards the proximal MTJ around the ischial tuberosity at a constant speed. This process was performed a few times for each participant until determining the correct path to visualise the BFlh fascicles. Then, the first measurement followed the visible path within the ultrasound gel on the skin. During the measurements, the assessor continuously adjusted the ultrasound probe to make the fascicles and aponeuroses of the BFlh visible based on different anatomical features of the BFlh (Fig 1). Then, four visible fascicles starting around the BFlh’s mid-belly were digitised using the ultrasound machine’s measure function. The four fascicle length values were averaged and accepted as the BFlh fascicle length. Subsequently, this process was repeated after totally removing the ultrasound probe from the skin after about two minutes of a waiting period. The mean value of the two measurements was accepted as the BFlh fascicle length of the measured leg.

#### 2.4.2. The BFlh mid-belly and distal MTJ passive stiffness measurements

**Fig 2:**
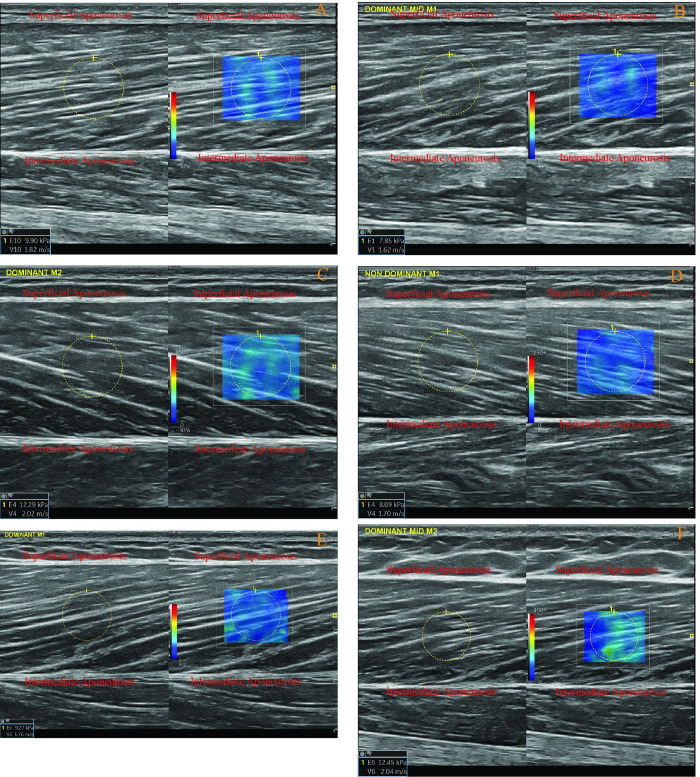
A-F: Random examples of the measurements of mid-muscle belly passive stiffness of the biceps femoris long head via ultrasound-based shear-wave elastography in various participants. The different image orientations, whether from left to right or vice versa, result from the 180° differences in the grip positions of the ultrasound probes during each measurement, which do not affect the outcomes.

**Fig 3:**
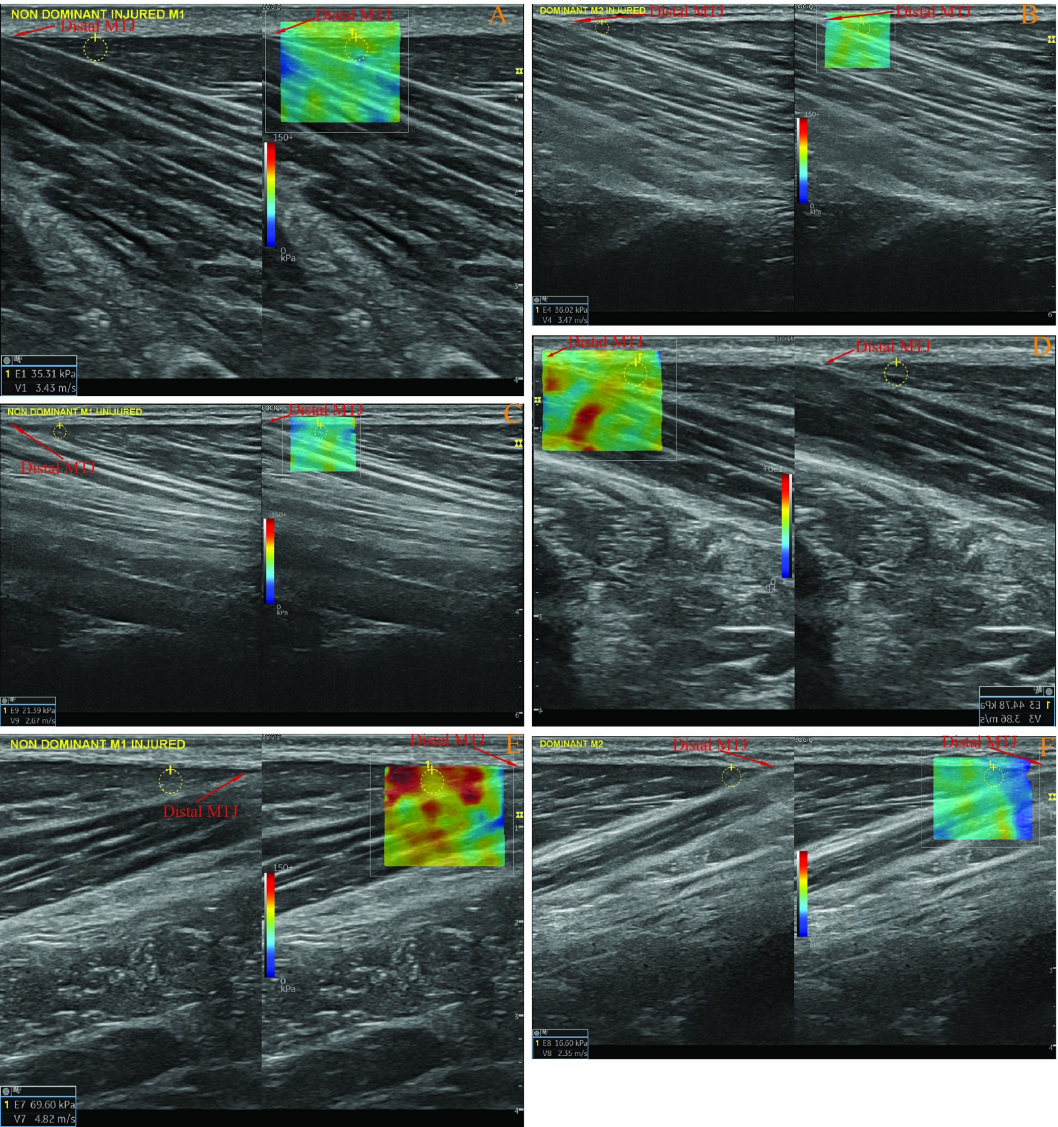
A-F: Random examples of the measurements of distal musculotendinous junction passive stiffness of the biceps femoris long head via ultrasound-based shear-wave elastography in various participants. The different image orientations, whether from left to right or vice versa, result from the 180° differences in the grip positions of the ultrasound probes during each measurement, which do not affect the outcomes.

Participants kept their position on a medical bed as described in the fascicle length measurement section for both mid-muscle belly and distal MTJ stiffness measurements of the BFlh. The ultrasound probe was placed on the 50% distance between the proximal MTJ and Distal MTJ on the muscle belly [39]. The probe was arranged parallel to the muscle fascicle orientations as described in the BFlh fascicle length measurement section. At the same time, the superficial and intermediate aponeuroses were visible, and they were parallel to each other as much as they could be at the location [39]. A slight and equal transducer pressure was applied during the measurements [39]. The region of interest (ROI) was selected on the centre of the BFlh muscle, and an elastogram with a 1 cm diameter was taken from the ROIs [39] (Fig 2). For the distal MTJ stiffness measurements of the BFlh, the participants kept the same position in the mid-muscle belly passive muscle stiffness of the BFlh. The ultrasound probe was oriented parallel to the BFlh muscle orientation, and the distal MTJ was visualised. The distal MTJ was determined by following the previous relevant research [40, 41]. An elastogram with a 0.25 cm diameter was taken from the closest place to the distal MTJ, where the 0.25 cm elastogram chamber fits into the triangle of the distal MTJ (Fig 3). The measurements were replicated by totally removing the ultrasound probe from the skin, and the mean value of these two measurements was accepted as the mid-belly passive muscle stiffness of the BFlh.

### 2.5. Statistical analyses

The BFlh fascicle length, mid-belly and distal MTJ passive stiffness results complied with parametric test assumptions (Levene’s test) and were compared between the injured legs, uninjured contralateral legs, and both legs of the control group via one-way analysis of variance (ANOVA). Additionally, the one-way ANOVA was also used to compare the participants’ physical characteristics and total years of performing sports to confirm no significant differences between comparison groups. Tukey posthoc tests were performed to assess statistical significance between grouped results using SPSS software (IBM SPSS Statistics, version 29). The alpha level was set as 0.05 for the statistical significance. Cohen’s *d*-effect sizes [42] were calculated for all parameters. Effect sizes for our sample consisting of highly trained athletes were interpreted as follows: < 0.25, trivial; 0.25–0.50, small; 0.50–1.00, moderate; > 1.00, large [43]. For intra-rater reliability analyses, the intraclass correlation coefficients (ICC, two-way mixed, absolute agreement on single measurements) were calculated for each outcome. The reliability results were classified as follows: < 0.50, low reliability; 0.50–0.75, moderate reliability; 0.75–0.90, high reliability; > 0.90, very high reliability [16].

## 3. Results

The injured group consisted of 8 professional runners, 8 semi-professional football players, 2 semi-professional basketball players, and 1 semi-professional cyclist. The control group consisted of the athletes who exactly matched with the injured group (8 professional runners, 8 semi-professional football players, 2 semi-professional basketball players, and 1 semi-professional cyclist). There were no significant differences between the participants’ physical characteristics and total years of performing sports between the groups (Table 1).

**Table 1:** Participants’ physical characteristics and total years of performing sport.

	Injured group (n = 19)	Control group (n = 19)	*p*-value for Levene’s test	*the p*-value for the mean difference
**Age (years)**	29.3 ± 6.5	26.4 ± 7.3	0.504	0.195
**Height (cm)**	171.2 ± 5.7	171.6 ± 6	0.844	0.825
**Weight (kg)**	68.9 ± 9.2	66.2 ± 8.6	0.958	0.358
**BMI (kg/m** ^**2**^ **)**	23.5 ± 2.9	22.5 ± 2.7	0.747	0.262
**Total years of performing the sport**	8.7 ± 5.5	8.4 ± 5.3	0.661	0.870

In the injured group, ten participants injured their dominant legs and nine participants injured their non-dominant legs. Four injuries were located around proximal MTJ, eight were located around distal MTJ, and seven were situated around the mid-muscle belly of the BFlh. Among the injured athletes, fourteen athletes experienced the HSI for the first time (mean time to injury = 9 ± 11.8 months ago), and five athletes experienced the HSI twice (mean time to first injury = 3.9 ± 2.1 years ago; mean time to last injury = 12.4 ± 14.8 months ago). In the injured group, three athletes stated that they have received a structured physiotherapy program; three athletes received massage; four athletes mentioned they received ice compression, one of the four athletes mentioned he swam in addition to the ice compression during the healing process; two athletes pointed out they used warm compression during the healing process; eight athletes mentioned they did not receive any rehabilitation and waited for the natural healing process.

All the measurements were performed twice except for one participant for the fascicle length and distal MTJ muscle stiffness measurements due to a human error in recording the results during the measurement sessions. Therefore, these measurements could not be included in the reliability analysis. However, the single measurements were accepted as the valid fascicle length and distal MTJ values in the primary analyses to compare injured and uninjured athletes. The reliability results of the measurements were as follows: Fascicle length (n = 75 legs), ICC = 0.982, 95% CI [0.971, 0.988]; mid-belly muscle stiffness (n = 76 legs), ICC = 0.973, 95% CI [0.955, 0.988], distal MTJ stiffness (n = 75 legs), ICC = 0.995, 95% CI [0.993, 0.997].

BFlh fascicle length measurements in the injured legs compared with the uninjured contralateral legs showed no significant difference (Mean difference (MD) = -0.43 ± 0.70 cm, *p* = 0.719). However, BFlh fascicle lengths of the injured legs in comparison with the legs of the control group revealed a trend to be shorter with a medium effect size despite its statistical nonsignificance (*d* = 0.62, MD = -1.10 ± 2.93 cm, *p* = 0.067) (Table 2). Moreover, the difference in the BFlh fascicle lengths between the uninjured contralateral legs of the injured athletes and both legs of the control group were not significantly different (MD = -0.67 ± 2.9 cm, *p* = 0.362) (Table 2). For the mid-belly passive muscle stiffness of the BFlh between the injured leg and control group legs, a significantly higher stiffness was detected for the injured legs (MD = 3.29 ± 6.09 KPa, *p* = 0.009) (Table 2). However, within injured athletes, the comparison of injured and uninjured legs showed no difference (MD = 2.17 ± 6.71, *p* = 0.198**)**. Moreover, the passive stiffness of the BFlh distal MTJ was significantly higher in the injured legs compared with the contralateral uninjured legs (10.97 ± 14.46, *p* = 0.017). These findings were consistent in further comparisons between the injured legs and both legs of the control group (MD = 20.49 ± 19.56, *p* < 0.001), and between the contralateral uninjured legs and both legs of the control group (MD = 9.52 ± 17.66, *p* = 0.017) (Table 2).

**Table 2:** Differences in the BFlh fascicle length, Mid-muscle belly stiffness and distal MTJ stiffness between the groups.

	Injured thighs (n = 19) (mean ± SD)	Uninjured contralateral thighs (n = 19) (mean ± SD)	Control thighs (n = 38) (mean ± SD	Mean Difference Cohen’s *d* effect size (Injured thighs vs. uninjured contralateral thighs) (mean ± SD, ES)	Mean Difference and Cohen’s *d* effect size (Injured thighs vs. control thighs) (mean ± SD, ES)	Mean Difference (Uninjured contralateral thighs vs. control thighs) (mean ± SD, ES)
**Fascicle length (cm)**	8.03 ± 2	8.46 ± 1.86	9.13 ± 1.52	-0.43 ± 0.7 *p* = 0.719, ***d* = 0.22**	-1.1 ± 2.93 *p* = 0.067, ***d* = 0.62**	-0.67 ± 2.9 *p* = 0.362, ***d* = 0.39**
**Passive stiffness of mid-muscle belly (kPa)**	13.2 ± 6.4	11.03 ± 3.5	9.91 ± 1.81	2.17 ± 6.71 *p* = 0.198, ***d* = 0.37**	3.29 ± 6.09 p* = 0.009, *d* = 0.7	1.12 ± 3.22 *p* = 0.557; ***d* = 0.4**
**Passive stiffness of distal musculotendinous junction (kPa)**	31.95 ± 17.39	20.98 ± 15.12	11.46 ± 5	10.97 ± 14.46 p* = 0.017, *d* = 0.67	20.49 ± 19.56 p** < 0.001, *d* = 1.6	9.52 ± 17.66 p* = 0.017, *d* = 0.85

Abbreviations. BFlh, biceps femoris long head; ES, effect size; MTJ, musculotendinous junction; SD, standard deviation. *, statistically significant (p < 0.05); **, statistically significant (p < 0.001).

## 4. Discussion

The structural parameters of human skeletal muscles have traditionally been given great attention by studies focusing on sports performance [44–67] and recently by research conducted on musculoskeletal injuries [12, 13, 16, 38, 68–76]. Likewise, this study has retrospectively examined athletes with and without HSI to compare changes in the muscle structure-related parameters of the BFlh muscle after an HSI. BFlh fascicle length was measured via b-mode panoramic ultrasound scanning, plus mid-muscle belly and distal muscle belly stiffness by ultrasound-based shear wave elastography.

This study employed a panoramic ultrasound scanning to measure the BFlh fascicle length which is methodologically different to former relevant retrospective studies [18–22] comparing athletes with and without HSI history. Most of these retrospective studies [18, 20–22] used the trigonometric equation technique, except for one study employing the manual linear extrapolation technique [19] for estimating the BFlh fascicle length. By using panoramic ultrasound scanning, all the lengths of BFlh fascicles could be monitored and calculated without an estimation of invisible parts, which may reduce an overestimation of the BFlh fascicles [17]. Only one former retrospective study [77] compared injured and uninjured legs of previously injured athletes using panoramic ultrasound scanning, however, they did have an external control (i.e., uninjured matched athletes). Our study did not detect significant differences between BFlh fascicle length despite being appropriately powered (0.96 statistical power). There were trivial and moderate effect sizes for the differences between the limbs of the injured group (MD = -0.43 ± 0.70 cm, *d* = 0.22) and between the injured leg of the injured group and legs of the controls (MD = -1.10 ± 2.93 cm, *d* = 0.62). This suggests that differences in BFlh between groups may be relevant for HSI susceptibility.

The previous retrospective studies found shorter BFlh fascicle lengths of the injured leg compared to the control ranging between -1.74 cm to -0.47 cm. In agreement, our study found a decrement of BFlh fascicle length (about -1.10 cm) in the injured legs of the athletes. Findings and the BFlh ultrasound assessment methods of the previous studies are presented in Table 3. A recent meta-analytic study [26] indicated moderately shorter BFlh fascicle lengths of injured legs of the athletes (effect size = 0.57), which is similar to our present research (effect size = 0.62). Variability of results in the literature might have multifactorial causes including athlete age [47, 78, 79], ethnicity [80], sports disciplines [81], rehabilitation process [82–84], abd ultrasound measurement methods [17, 38]. However, due to the nature of the retrospective study, longitudinal trials are needed to understand the differences in athletes after HSI.

**Table 3:** Differences in the BFlh fascicle lengths between athletes with and without hamstring strain injury history in the previous retrospective studies.

Study	Participants	Injured leg vs. uninjured contralateral leg mean difference (cm) and ES	Injured leg vs. control group mean difference (cm) and ES	Ultrasound assessment method
**de Lima-E-Silva et al. [18]**	20 injured football players vs. 60 uninjured football players	0.11 cm (*p* = 0.91, *d* = 0.04)	-1.80 cm (*p* = 0.02*, *d* = 0.59)	Trigonometric equation method
**Nin et al. [19]**	5 injured athletes (mix) vs. 10 uninjured athletes (mix)	-0.41 cm (*p* = NG, *d* = -0.37)	-0.58 cm (*p* = NG, *d* = -0.67)	Manual Linear Extrapolation method
**Pimenta et al. [20] (Baseline values)**	12 injured football players vs. 20 uninjured football players:	-1.74 cm (*p* = 0.012*, *d* = NG)	-0.80 cm (*p* = 0.014*, *d* = NG)	Linear Extrapolation method via trigonometric equation for invisible part
**Timmins et al. [21]**	12 injured Australian footballers vs. 18 uninjured Australian footballers	-0.47 cm (*p* = NG, *d* = NG)	-0.73 cm (*p* = NG, *d* = NG)	Trigonometric equation method
**Timmins et al. [22]**	16 previously injured athletes vs. 20 recreationally active controls	-1.54 cm (p < 0.001*, *d* = -0.74)	-0.47 cm (The control group was accepted as the average results of both limbs (*p* = NG, *d* = NG)	Trigonometric equation method

Abbreviations. BFlh, biceps femoris long head; ES, effect size; NG, not given; *, statistically significant (p < 0.05).

A previous study [26] suggested that neural inhibition following the injury [24–29] and reduced exertion may cause BFlh fascicle shortening at the early phase of rehabilitation [22]. Together with the former, decreased participation in sports [24] are a possible underlying mechanism for the shortening of BFlh fascicle lengths after injury. Future studies are needed to monitor the architecture of the hamstrings before the HSI, during rehabilitation, and after returning to play for a better understanding of the mechanisms behind the architectural alterations after the HSI. Based on the findings of this study, it may be suggested that rehabilitation programs should include exercises that can lead to fascicle lengthening in the hamstrings of both legs of previously injured athletes to minimise the possibility of microscopic damage of the fascicles during eccentric contraction [25, 26, 85].

To the authors’ knowledge, this study was the first retrospective study comparing the passive distal MTJ stiffness of the BFlh between athletes with and without an HSI history. Our study observed significantly higher stiffness in the previously injured BFlh muscles at the mid-muscle belly (MD = 3.29 ± 6.09 kPa, *p* = 0.009, *d* = 0.70) and distal MTJ compared to the controls (MD = 20.49 ± 19.56 kPa, *p* < 0.001, *d* = 1.60) with moderate and large effect sizes, respectively. A previous retrospective study [19] pointed out larger stiffness (*d* = 1.28) at the muscle belly of the BFlh in HSI athletes compared to controls. Our study also detected greater mid-belly stiffness, though only moderately higher (*d* = 0.70), in the athletes with HSI history. Additionally, we detected large (*d* = 1.60) differences at the distal MTJ of the BFlh between the injured muscle and controls. Additionally, we foundmoderately higher stiffnesses at the distal MTJ of this injured leg compared to the contralateral legs (*d* = 0.67**)**. The larger stiffness at the distal MTJ (*d* = 1.60) compared to the mid-part (*d* = 0.70) of the injured BFlh can be caused by distal MTJ injuries which were the most frequent in our sample (8 of 19 athletes). Distal MTJ injuries were pointed out as common [9], more recurrent and severe, and requiring longer rehabilitation [9, 10]. Reasons for higher stiffness in the injured legs compared with the controls might be explained by potential scarring of tissue around the injury location [24, 26, 30–34]. Tissue changes (i.e., mechanical properties) would affect the passive tension and stiffness of the BFlh structure towards higher values [86]. Moreover, sixteen out of nineteen of the injured athletes in our study did not receive a structured physiotherapy program including eccentric training and static stretching. Such structured rehabilitation may help to prevent muscle shortening [39] and improve muscle elasticity [87] during the healing process. Higher stiffness after lower extremity injuries is not uncommon. A previous study detected considerable increments in hamstrings’ passive muscle stiffness for both injured and uninjured legs after anterior cruciate ligament reconstructions compared to healthy controls [35]. Another study [34] observed significant increases in stiffness within the first 6 weeks and 6 months after a tendon injury. Of our participants, ten were injured within less than six months, with seven of them injured approximately six weeks (1–2 months) before the measurement day (Supporting Information S1). Measurements closer to the injury occurrence may have contributed to the differences in the distal MTJ stiffness results.

Moreover, it is documented in the literature that hamstring muscle stiffness results may be influenced by age [88, 89], genetics [88, 90], sex [91], sports profession [39, 92], pelvic tilt type [93], hip and knee positions [94], injury and scar status [32–34], and operator of the shear-wave elastography [95]. Besides reasons based on the characteristics and history of the individual, stiffness results of the BFlh vary between different ultrasound brands and versions even at similar measurement positions in healthy participants. Baseline or reliability values of the BFlh passive muscle belly stiffness at resting appeared as 4.5 kPa [96], 9.91 kPa (this study), 10.54–15.72 [97], 10.8 kPa [98], 11.3–11.7 kPa [99], 11.57 kPa, [100], 14.43–16.27 kPa [101], 15.74–19.01 kPa [102], 16.47–19.87 kPa [41], 16.9–24.7 kPa [39]. A future study should compare differences in the BFlh stiffness values on the same sample between commercially available devices to determine the comparability of results in the literature.

Outcomes suggest rehabilitation programmes should consider establishing muscle elasticity after the HSI. In one study, researchers found a four-week static stretching program was effective in maintaining elasticity of the BFlh [87]. However, the effectiveness of other rehabilitation modalities are not well-documented in the literature. Future studies should investigate the effectiveness of rehabilitation modalities on the stiffness of hamstrings. Ultrasound-based shear-wave elastography could be a useful tool to monitor muscle stiffness during the rehabilitation process in the injured area.

The first limitation of this study is its retrospective cross-sectional design which does not allow conclusions towards cause or effect of the measured parameters for the HSI. Another limitation of the retrospective design is not having detailed information about the participants’ rehabilitation process, which may lead to difficulties in interpreting the results. Additionally, the absence of retrospective information regarding the severity of injuries in the players with a history of HSI is acknowledged as a limitation in this study. Moreover, this study only applied intra-rater reliability assessments, which is another confounding factor. The measurements could not blinded due to the multiple required tasks of the assessor during the data collection. Furthermore, another confounding factor of this study was the BFlh fascicle length measurement method. There is no gold standard measurement method for BFlh fascicle length in the literature [17]. Despite this, panoramic ultrasound scanning does not overestimate the BFlh fascicle length compared to trigonometric equations and manual linear extrapolation methods. A recent study [103] stated that ultrasound assessment can overestimate increases in serial sarcomere numbers by about 5%. On the other hand, shear-wave elastography values can be influenced by assessment depths and the size of the ROIs and elastograms [104]. Despite the potential advantages of measuring a specific location within a muscle, not assessing the entire muscle and tendon structures can be another drawback of ultrasound-based shear-wave studies, such as ours. Therefore, future studies should aim to provide standardised measurement approaches for measuring the skeletal muscles and tendons at their entire muscle volumes by providing specific location and overall volume stiffness information.

Initially, it was also planned to measure the proximal MTJ stiffness of the BFlh as an additional study outcome. However, the shear waves of the ultrasound machine could not reach a full ROI colour distribution at the proximal MTJ of the BFlh. This issue might have been because: a) the shear waves of our ultrasound machine were not durable enough to pass through gluteus maximus muscle fascicles, which have a different orientation than the BFlh, for reaching the proximal MTJ of the BFlh; b) the higher depth of the proximal MTJ compared the distal MTJ, c) the technical abilities of the ultrasound machine, or d) another reason which may be identified by future studies. Consequently, we decided not to continue proximal MTJ stiffness measurements after a few trials, and we noted this issue as a limitation of this study.

## 5. Conclusions

This study has used relatively advanced measurement methods for measuring the BFlh fascicles and stiffness, e.g. panoramic ultrasound scanning and shear-wave elastography. Results showed significantly higher mid-muscle belly and distal MTJ stiffness values in previously injured athletes compared to the controls. Despite being only a trend, moderately shorter fascicles were observed in the injured players compared to the controls. Physical therapy and rehabilitation programmes should aim to improve BFlh muscle elasticity and fascicle length after the HSI. Future prospective studies should investigate whether higher stiffness is a cause or consequence of HSI.

## Supporting information

S1 Data (PDF)
