# Supplementary material for: A retrospective comparison of the biceps femoris long head muscle structure in athletes with and without hamstring strain injury history
Source: PLoS One. 2024 Feb 26;19(2):e0298146. doi: 10.1371/journal.pone.0298146 (PMC10896514; doi:10.1371/journal.pone.0298146)
Supplement: S1 Data — (PDF) [file pone.0298146.s001.pdf]

Group	IFL	MID_SWE	SWE_DIST/	RELIABILITY	RELIABILITY	RELIABILITY	RELIABILITY	RELIABILITY
1.00	7.76	7.42	20.80	9.17	9.21	7.57	7.95	8.01
1.00	12.08	11.28	49.33		12.08	11.81	11.99	11.27
1.00	7.54	9.69	36.30	8.30	8.35	7.91	7.16	9.15
1.00	9.99	36.37	20.58	10.10	9.88	11.13	11.35	35.50
1.00	7.40	11.27	39.98	6.93	7.14	7.36	7.44	10.28
1.00	6.47	9.85	18.43	6.37	6.57	8.27	8.49	10.43
1.00	8.53	12.10	8.95	8.61	8.44	8.70	8.54	12.15
1.00	7.87	11.02	17.16	7.80	7.94	8.77	9.27	10.58
1.00	8.37	10.76	10.86	7.64	7.98	8.36	8.38	8.39
1.00	7.83	11.27	26.18	7.74	7.92	8.39	8.02	10.88
1.00	6.78	10.37	52.06	7.12	7.73	7.02	6.54	9.62
1.00	9.32	9.97	8.31	9.25	9.39	8.51	8.84	10.15
1.00	9.86	20.52	30.11	9.80	9.65	9.95	9.77	12.62
1.00	11.84	13.60	37.43	11.82	11.86	11.80	12.19	12.28
1.00	8.64	10.62	41.06	8.39	8.89	8.68	9.00	9.92
1.00	5.37	18.05	69.90	6.35	6.33	5.38	5.36	15.09
1.00	5.11	10.27	29.79	5.40	5.20	5.11	5.11	8.13
1.00	4.98	15.54	61.31	5.03	4.94	5.88	5.69	15.42
1.00	6.80	10.95	28.65	7.24	6.82	6.89	6.72	9.66
2.00	9.19	8.48	7.50	7.63	7.64	7.63	7.75	10.74
2.00	11.90	15.02	33.23	7.57	7.26	7.34	7.61	10.68
2.00	8.32	9.40	11.02	7.70	7.81	8.16	7.74	12.80
2.00	11.24	11.93	12.85	7.52	7.39	7.58	7.66	9.84
2.00	7.03	11.30	21.59	7.53	7.31	7.62	7.54	11.99
2.00	8.38	10.44	16.09	9.83	10.16	10.39	10.40	8.51
2.00	8.62	7.83	7.62	10.51	11.31	9.04	9.38	9.27
2.00	9.02	10.53	7.90	8.31	9.10	8.51	8.81	8.94
2.00	7.81	8.21	9.78	11.25	11.83	11.45	12.51	8.32
2.00	8.21	22.28	14.36	11.89	12.07	10.85	10.88	10.94
2.00	7.42	9.33	12.17	6.82	6.97	7.71	7.84	5.59
2.00	8.68	7.59	8.73	8.39	8.77	8.95	8.94	10.54
2.00	9.72	12.24	18.71	10.27	9.54	9.84	9.61	11.26
2.00	11.99	10.09	22.60	8.50	8.38	7.59	7.64	12.33
2.00	8.84	12.33	24.02	8.92	8.58	8.90	9.42	7.90
2.00	6.34	15.21	43.96	9.16	9.18	9.24	9.42	10.88
2.00	5.30	7.86	62.61	12.47	12.00	12.21	11.86	7.79
2.00	5.78	9.66	44.72	10.33	10.12	9.17	9.09	9.10
2.00	7.03	9.91	19.25	8.81	8.99	9.90	10.05	8.75
3.00	7.63	11.42	12.07					
3.00	7.42	11.03	7.97					
3.00	7.76	12.98	8.07					
3.00	7.45	10.23	6.62					
3.00	7.42	11.58	11.11					
3.00	10.00	8.66	21.06					
3.00	10.91	9.27	24.22					
3.00	8.71	9.05	15.81					

3.00	11.54	8.75	13.03
3.00	11.98	11.85	12.42
3.00	6.89	5.61	18.36
3.00	8.58	10.68	7.04
3.00	9.91	11.78	13.53
3.00	8.44	12.16	15.26
3.00	8.75	8.22	10.74
3.00	9.17	10.51	7.48
3.00	12.23	8.01	6.90
3.00	10.22	8.91	7.16
3.00	8.90	8.78	16.29
3.00	7.69	9.19	19.94
3.00	7.48	8.44	6.40
3.00	7.95	9.19	8.51
3.00	7.62	13.21	8.84
3.00	7.58	9.26	11.10
3.00	10.40	9.93	21.14
3.00	9.21	8.82	14.32
3.00	8.66	10.01	19.20
3.00	11.98	8.42	8.09
3.00	10.86	12.98	7.24
3.00	7.78	6.63	5.36
3.00	8.94	13.23	5.52
3.00	9.73	10.72	12.62
3.00	7.61	11.85	9.55
3.00	9.16	10.86	10.88
3.00	9.33	8.92	8.25
3.00	12.04	7.87	7.45
3.00	9.13	9.22	6.43
3.00	9.97	8.53	9.86

RELIABILIT	RELIABILIT	RELIABILIT	RELIABILIT	RELIABILIT	RELIABILIT	RELIABILIT	OVERALL_F	OVERALL_F
8.94	7.80	7.04	8.52	6.47	20.30	21.29	9.17	9.21
11.28	15.84	14.19	50.28	48.37	33.73	32.72		12.08
9.65	9.02	10.35	10.66	11.37	35.31	37.28	8.30	8.35
37.23	12.28	11.58	20.68	20.47	12.62	13.08	10.10	9.88
12.31	11.29	11.25	21.19	21.98	38.43	41.52	6.93	7.14
9.26	9.87	11.01	18.48	18.37	16.01	16.17	6.37	6.57
12.04	7.35	8.31	8.96	8.94	7.14	8.10	8.61	8.44
11.46	10.63	10.43	17.24	17.07	7.93	7.86	7.80	7.94
8.02	10.83	10.69	9.18	10.37	11.43	10.29	7.64	7.98
11.65	21.35	23.21	29.59	22.77	14.46	14.26	7.74	7.92
9.03	9.63	11.10	12.99	11.34	50.02	54.09	7.12	7.73
9.78	6.56	8.61	8.63	7.99	8.88	8.58	9.25	9.39
11.86	19.64	21.40	18.96	18.45	30.73	29.49	9.80	9.65
14.92	10.04	10.14	38.84	36.02	21.39	23.80	11.82	11.86
11.32	12.11	12.55	39.85	42.27	23.67	24.36	8.39	8.89
15.33	18.35	17.74	43.82	44.10	70.19	69.60	6.35	6.33
7.58	10.99	9.54	62.58	62.64	29.38	30.20	5.40	5.20
15.66	9.49	9.82	61.03	61.59	44.78	44.65	5.03	4.94
10.16	10.60	11.30	28.96	28.33	18.73	19.77	7.24	6.82
12.09	8.62	9.76	11.44	12.69	19.90	19.98	7.63	7.64
11.37	8.63	8.24	8.17	7.77	6.15	6.65	7.57	7.26
13.15	10.07	8.30	8.50	7.64	8.83	8.19	7.70	7.81
10.61	13.64	12.78	6.58	6.66	8.82	8.86	7.52	7.39
11.17	9.47	9.04	11.17	11.04	11.10		7.53	7.31
8.80	9.90	9.96	21.13	20.99	21.13	21.14	9.83	10.16
9.26	8.69	8.94	22.80	25.63	14.66	13.97	10.51	11.31
9.16	10.12	9.90	15.02	16.60	19.98	18.41	8.31	9.10
9.18	8.29	8.54	13.42	12.63	8.34	7.83	11.25	11.83
12.75	12.25	13.71	13.32	11.51	6.73	7.74	11.89	12.07
5.63	6.28	6.97	17.94	18.78	5.50	5.22	6.82	6.97
10.81	13.63	12.82	6.13	7.95	5.90	5.14	8.39	8.77
12.29	11.28	10.16	13.90	13.15	12.81	12.42	10.27	9.54
11.99	11.66	12.04	15.85	14.67	9.65	9.45	8.50	8.38
8.54	10.88	10.83	10.84	10.63	10.89	10.86	8.92	8.58
10.14	8.53	9.31	7.16	7.79	8.04	8.46	9.16	9.18
8.23	7.46	8.27	7.11	6.69	7.05	7.85	12.47	12.00
8.72	9.20	9.23	7.20	7.12	6.82	6.03	10.33	10.12
8.80	8.30	8.76	15.91	16.66	9.85	9.87	8.81	8.99
							7.57	7.95
							11.81	11.99
							7.91	7.16
							11.13	11.35
							7.36	7.44
							8.27	8.49
							8.70	8.54
							8.77	9.27

8.36	8.38
8.39	8.02
7.02	6.54
8.51	8.84
9.95	9.77
11.80	12.19
8.68	9.00
5.38	5.36
5.11	5.11
5.88	5.69
6.89	6.72
7.63	7.75
7.34	7.61
8.16	7.74
7.58	7.66
7.62	7.54
10.39	10.40
9.04	9.38
8.51	8.81
11.45	12.51
10.85	10.88
7.71	7.84
8.95	8.94
9.84	9.61
7.59	7.64
8.90	9.42
9.24	9.42
12.21	11.86
9.17	9.09
9.90	10.05

OVERALL_F	OVERALL_F	OVERALL_F	OVERALL_REL_MTJ_2
8.01	8.94	8.52	6.47
11.27	11.28	50.28	48.37
9.15	9.65	10.66	11.37
35.50	37.23	20.68	20.47
10.28	12.31	21.19	21.98
10.43	9.26	18.48	18.37
12.15	12.04	8.96	8.94
10.58	11.46	17.24	17.07
8.39	8.02	9.18	10.37
10.88	11.65	29.59	22.77
9.62	9.03	12.99	11.34
10.15	9.78	8.63	7.99
12.62	11.86	18.96	18.45
12.28	14.92	38.84	36.02
9.92	11.32	39.85	42.27
15.09	15.33	43.82	44.10
8.13	7.58	62.58	62.64
15.42	15.66	61.03	61.59
9.66	10.16	28.96	28.33
10.74	12.09	11.44	12.69
10.68	11.37	8.17	7.77
12.80	13.15	8.50	7.64
9.84	10.61	6.58	6.66
11.99	11.17	11.17	11.04
8.51	8.80	21.13	20.99
9.27	9.26	22.80	25.63
8.94	9.16	15.02	16.60
8.32	9.18	13.42	12.63
10.94	12.75	13.32	11.51
5.59	5.63	17.94	18.78
10.54	10.81	6.13	7.95
11.26	12.29	13.90	13.15
12.33	11.99	15.85	14.67
7.90	8.54	10.84	10.63
10.88	10.14	7.16	7.79
7.79	8.23	7.11	6.69
9.10	8.72	7.20	7.12
8.75	8.80	15.91	16.66
7.80	7.04	20.30	21.29
15.84	14.19	33.73	32.72
9.02	10.35	35.31	37.28
12.28	11.58	12.62	13.08
11.29	11.25	38.43	41.52
9.87	11.01	16.01	16.17
7.35	8.31	7.14	8.10
10.63	10.43	7.93	7.86

10.83	10.69	11.43	10.29
21.35	23.21	14.46	14.26
9.63	11.10	50.02	54.09
6.56	8.61	8.88	8.58
19.64	21.40	30.73	29.49
10.04	10.14	21.39	23.80
12.11	12.55	23.67	24.36
18.35	17.74	70.19	69.60
10.99	9.54	29.38	30.20
9.49	9.82	44.78	44.65
10.60	11.30	18.73	19.77
8.62	9.76	19.90	19.98
8.63	8.24	6.15	6.65
10.07	8.30	8.83	8.19
13.64	12.78	8.82	8.86
9.47	9.04	11.10	
9.90	9.96	21.13	21.14
8.69	8.94	14.66	13.97
10.12	9.90	19.98	18.41
8.29	8.54	8.34	7.83
12.25	13.71	6.73	7.74
6.28	6.97	5.50	5.22
13.63	12.82	5.90	5.14
11.28	10.16	12.81	12.42
11.66	12.04	9.65	9.45
10.88	10.83	10.89	10.86
8.53	9.31	8.04	8.46
7.46	8.27	7.05	7.85
9.20	9.23	6.82	6.03
8.30	8.76	9.85	9.87
